# A New Morphological Type of *Volvox* from Japanese Large Lakes and Recent Divergence of this Type and *V*. *ferrisii* in Two Different Freshwater Habitats

**DOI:** 10.1371/journal.pone.0167148

**Published:** 2016-11-23

**Authors:** Hisayoshi Nozaki, Noriko Ueki, Nanako Isaka, Tokiko Saigo, Kayoko Yamamoto, Ryo Matsuzaki, Fumio Takahashi, Ken-ichi Wakabayashi, Masanobu Kawachi

**Affiliations:** 1 Department of Biological Sciences, Graduate School of Science, University of Tokyo, Hongo 7-3-1, Bunkyo-ku, Tokyo, 113–0033, Japan; 2 Laboratory for Chemistry and Life Science, Institute of Innovative Research, Tokyo Institute of Technology, Yokohama, 226–8503, Japan; 3 Center for Environmental Biology and Ecosystem Studies, National Institute for Environmental Studies, Onogawa 16–2, Tsukuba-shi, Ibaraki, 305–8506, Japan; 4 College of Life Sciences, Ritsumeikan University, Nojihigashi 1-1-1, Kusatsu-shi, Shiga, 525–8577, Japan; Donald Danforth Plant Science Center, UNITED STATES

## Abstract

*Volvox* sect. *Volvox* is characterized by having unique morphological characteristics, such as thick cytoplasmic bridges between adult somatic cells in the spheroids and spiny zygote walls. Species of this section are found from various freshwater habitats. Recently, three species of *Volvox* sect. *Volvox* originating from rice paddies and a marsh were studied taxonomically based on molecular and morphological data of cultured materials. However, taxonomic studies have not been performed on cultured materials of this section originating from large lake water bodies. We studied a new morphological type of *Volvox* sect. *Volvox* (“*Volvox* sp. Sagami”), using cultured materials originating from two large lakes and a pond in Japan. *Volvox* sp. Sagami produced monoecious sexual spheroids and may represent a new morphological species; it could be clearly distinguished from all previously described monoecious species of *Volvox* sect. *Volvox* by its small number of eggs or zygotes (5–25) in sexual spheroids, with short acute spines (up to 3 μm long) on the zygote walls and elongated anterior somatic cells in asexual spheroids. Based on sequences of internal transcribed spacer (ITS) regions of nuclear ribosomal DNA (rDNA; ITS-1, 5.8S rDNA and ITS-2) and plastid genes, however, the *Volvox* sp. Sagami lineage and its sister lineage (the monoecious species *V*. *ferrisii*) showed very small genetic differences, which correspond to the variation within a single biological species in other volvocalean algae. Since *V*. *ferrisii* was different from *Volvox* sp. Sagami, by having approximately 100–200 zygotes in the sexual spheroids and long spines (6–8.5 μm long) on the zygote walls, as well as growing in Japanese rice paddies, these two morphologically distinct lineages might have diverged rapidly in the two different freshwater habitats. In addition, the swimming velocity during phototaxis of *Volvox* sp. Sagami spheroids originating from large lakes was significantly higher than that of *V*. *ferrisii* originating from rice paddies, suggesting adaptation of *Volvox* sp. Sagami to large water bodies.

## Introduction

*Volvox* represents the most advanced member of volvocine green algae [1]. This genus was originally described by Linnaeus [2]. Smith [3] classified the genus *Volvox* into four sections (subdivisions of genus or taxa between genus and species) based on the differences in morphology of gelatinous matrix and cytoplasmic bridges between adult cells in spheroids. Recently, a new taxonomic system at section level was proposed to subdivide the genus into four monophyletic sections [4], in which taxonomic status of *Volvox* sect. *Volvox* (= *Euvolvox* sensu Smith [3]) has been unchanged [3, 4]. This section has unique morphological characteristics, such as thick cytoplasmic bridges between adult somatic cells and spiny zygote walls [3, 4]. *Volvox* sect. *Volvox* species constitute a clade that is separated from other sections of the genus *Volvox* [4–6].

Eight species have been recognized in the *Volvox* sect. *Volvox*, based on morphological characteristics of field-collected material [3, 7, 8]. Recently, based on morphological and molecular data of cultured materials of this section, two new species (*V*. *ferrisii* and *V*. *kirkiorum*) were found in Japanese rice paddies [8], and *V*. *capensis* was reported to originate from a marsh in Montana, USA [9]. However, species of *Volvox* sect. *Volvox* occurring as phytoplanktons in large lakes have not been studied, based on morphological and molecular data of cultured material.

The present study was undertaken to examine a new morphological type of *Volvox* sect. *Volvox* [“*Volvox* sp. Sagami”] occurring as phytoplanktons in large lakes and in a pond in Japan, based on combined analyses of molecular and morphological data from cultured material. Although *Volvox* sp. Sagami is closely related to *V*. *ferrisii* on the basis of molecular phylogenetic analyses, differences in morphological characteristics and habitats suggest rapid diversification of these two lineages in two different Japanese freshwater habitats.

## Materials and Methods

### Ethics statement

We collected *Volvox* sp. Sagami from two large lakes and a pond. Collection locations and details are shown in S1 Table. Collection of volvocalean algae in Lake Sagami and Lake Tsukui was permitted by the Tanigahara Water Purification Plant of Kanagawa Prefecture Companies Authority, the Kanagawa Prefectural Government, Japan. Collection of *Volvox* species was permitted in the Miyaike Pond by the Fumon Residents Association, Otsu, Shiga, Japan.

### Establishment of cultures and light microscopic observations

Water samples were collected from two large lakes and a pond (S1 Fig; S1 Table). Clonal cultures of *Volvox* sp. Sagami were established from the water samples in Petri dishes (90 × 20 mm), using the pipette-washing method [10]. The cultures were maintained in screw-cap tubes (18 × 150 mm) containing 11 mL artificial freshwater-6 (AF-6) [11] or AF-6/3 medium (AF-6 medium diluted with two volumes of distilled water [9]) at 20°C, 23°C, or 25°C on a 14 h light:10 h dark schedule under cool-white fluorescent lamps at an intensity of 80–130 μmol∙m^−2^∙s^−1^. The new wild strains of *Volvox* sp. Sagami (S1 Table) are available from Microbial Culture Collection at the Institute for National Environmental Studies [11] (http://mcc.nies.go.jp/localeAction.do;jsessionid=902B594FE29F71E317167414B91E6152?lang=en) as NIES-4021~4028 (S1 Table). To observe the morphology of asexual spheroids, the cultures were grown in Volvox thiamin acetate (VTAC) medium, containing 200 mg L^−1^ sodium acetate 4H_2_O [9, 11, 12], or VTAC/3 (VTAC medium diluted with two volumes of distilled water) at 25°C on 14:10 LD. A small aliquot of asexual spheroids, in actively grown 2- to 5-day-old cultures in tubes or Petri dishes (55 × 15 mm), was examined. Sexual spheroids developed spontaneously in a culture that was several days old, with VTAC/3 medium at 25°C on 14:10 LD, except for *Volvox* sp. Sagami strain 15-Sagami12-2, which did not produce sexual spheroids under those culture conditions. To enhance the production of sexual spheroids, urea soil Volvox thiamin/3 (USVT/3) medium [USVT medium (VTAC medium supplemented with 40 mg L^−1^ urea and 40 mL L^−1^ soil extract medium [9]) diluted with two volumes of distilled water] was also used. For maturation of sexual spheroids and the fertilized eggs or zygotes, 0.5–1.0 mL actively growing culture with sexual spheroids was inoculated into 11 mL USVT/3 medium.

Light microscopy was performed on a BX60 microscope (Olympus, Tokyo, Japan) equipped with Nomarski optics. The cells in spheroids were counted as described previously [3, 13]. Individual cellular sheaths of the gelatinous matrix of the spheroids were examined after mixing approximately 10 μL cultured material with 2–5 μL 0.002% (w/v in distilled water) methylene blue (1B-429 WALDECK GmbH & Co Division Chroma, Münster, Germany).

### Molecular experiments

To determine the phylogenetic position of the alga, we used the large subunit of Rubisco (*rbc*L), plus the photosystem II CP43 apoprotein (*psb*C) genes, and the internal transcribed spacer (ITS) regions of nuclear ribosomal DNA (rDNA; ITS-1, 5.8S rDNA, and ITS-2) from the operational taxonomic units (OTUs) or species/lineages listed in S1 Table. The two data sets (*rbc*L-*psb*C genes and ITS rDNA) were aligned as described previously [8, 9]. The alignments are available from TreeBASE: http://www.treebase.org/treebase-web/home.html; study ID: 20048). Designation of the outgroup or root was performed based on previous phylogenetic results [8, 9]. Maximum-likelihood (ML) analyses, based on the selected models (GTR+G and K2+G models for *rbc*L-*psb*C genes and ITS rDNA, respectively) with 1000 replicates of bootstrap analyses [14], were performed using MEGA 5.2.2 [15]. In addition, 1000 replicates of bootstrap analyses were performed by the maximum-parsimony (MP) method, based on a branch-and-bound search by PAUP 4.0b10 [16]. The secondary structures of ITS-2 were predicted as described previously [4, 9]. Nucleotide sequences of a potential group I intron inserted in the *Volvox* sp. Sagami *psb*C gene were determined by direct sequencing of PCR products, amplified by four new primers (S2 Table). The secondary structure map of group I introns was constructed as previously described [17].

### Swimming velocity during phototaxis

To measure swimming velocity during phototaxis, four strains of *Volvox ferrisii* were obtained from the Microbial Culture Collection at the Institute for National Environmental Studies for comparison with five *Volvox* sp. Sagami strains (S3 Table). They were cultured in screw-cap tubes containing 11 mL AF-6 or AF-6/3 media at 25°C, as described above.

Actively growing cultured materials 8 to 9 days old were used in the experiments. Approximately 1 mL cultured materials were placed in 24-wells plates (Becton, Dickinson and Company, NJ, USA), and observed under an SMZ1000 stereomicroscope (Nikon, Tokyo, Japan). Spheroids were illuminated with a green light-emitting diode (LED) (λ = 525 nm, <100 μmol photons·m^−2^·s^−1^) [18] from one side, observed under red light and video-recorded with an EOS Kiss X7 digital camera (Canon, Tokyo, Japan). Swimming velocities of 20 spheroids for each culture were obtained from the tracks for 3 s, following illumination with a green LED for 22 s using Image Hyper software (Science Eye, Saitama, Japan), and averaged. Approximate diameters in each culture were measured based on 32–45 randomly selected asexual spheroids, using ImageJ 1.48v software (National Institutes of Health, Bethesda, MD, USA).

## Results

### Morphology of asexual and sexual spheroids

**Fig 1:**
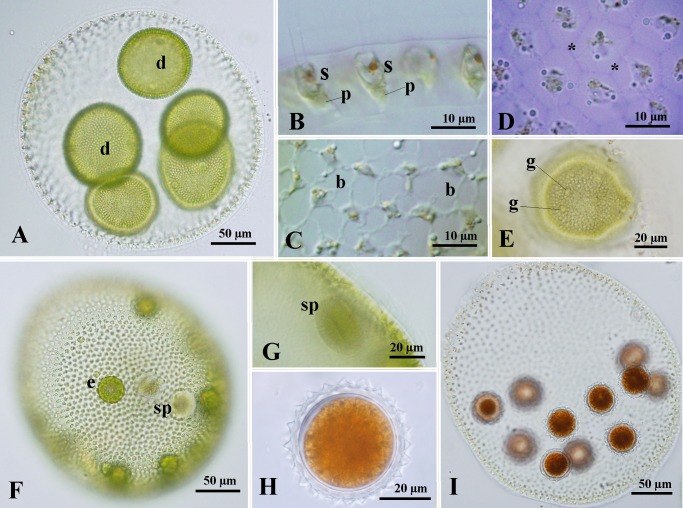
**Light microscopy of *Volvox* sp. Sagami strains 13-614-Vx13 (A-C), 13-614-Vx15 (D-G) and 14-614-Vx04 (H and I).** (A) Asexual spheroid with daughter colonies (d). (B-E) Part of asexual spheroids. (B) Side view of anterior cells, showing elongate-ellipsoidal or spindle cell shape, stigma (s) and pyrenoid (p) in the chloroplast. (C) Top view of somatic cells interconnected by cytoplasmic bridges (b). (D) Optical section of top view of cells surrounded by individual sheaths (asterisks). Stained with methylene blue. (E) Optical section of developing embryo during late stage of inversion. Note that gonidia (g) of the next generation are evident. (F) Monoecious sexual spheroid with eggs (e) and sperm packets (sp). (G) Side view of sperm packet (sp) in monoecious sexual spheroid. (H) Mature zygote with short and acute spines developing on the walls. (I) Sexual spheroid with mature zygotes.

Asexual spheroids of all *Volvox* sp. Sagami strains were spherical to pear-shaped, measured up to 590 μm long, and contained 2,000–10,000 (usually 2,500–5,000) biflagellate somatic cells and 4–8 gonidia distributed in the posterior two-thirds portion (Fig 1A). Somatic cells in the anterior pole of the spheroid were elongate-ellipsoidal or spindle-shaped in a side view, measuring up to 16 μm long (Fig 1B). Somatic cells have a cup-shaped chloroplast, with a single pyrenoid and a stigma (Fig 1B). The cells were connected via thick cytoplasmic bridges and enclosed by individual sheaths (Fig 1C and 1D). Gonidia were evident in juvenile spheroids or even during the late inversion stage of daughter spheroid formation (Fig 1E).

Although *Volvox* sp. Sagami strain 15-Sagami12-2 did not produce sexual spheroids under the present culture conditions, sexual spheroids in all other strains of *Volvox* sp. Sagami exhibited essentially the same morphological characteristics falling within the ranges described below.

Sexual spheroids were spherical, pear-shaped, or ellipsoidal with 4,000–8,000 cells and monoecious with 5–25 (usually 10–20) eggs and 1–5 sperm packets, and up to 500 μm long (Fig 1F). Sperm packets were compressed globoids composed of biflagellate male gametes (Fig 1G). Mature zygotes had a thick cell wall with spines, measuring 37–48 μm in diameter (without spines; Fig 1H and 1I). Spines of zygote walls were almost straight, with an acute tip, and measured up to 3 μm (Fig 1H).

### Molecular phylogeny

**Fig 2:**
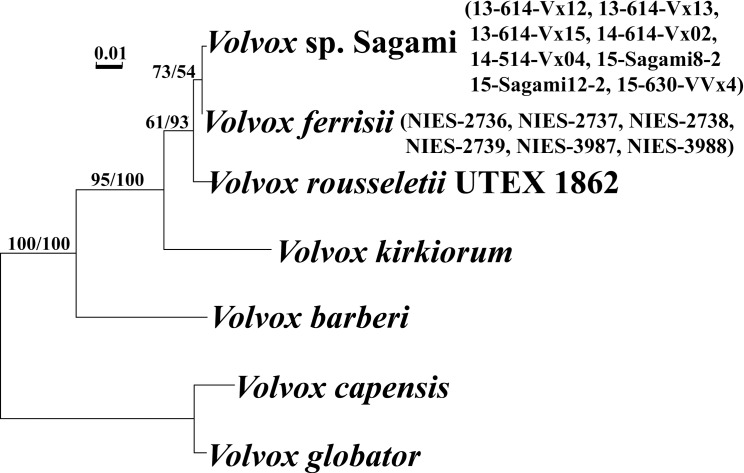
Maximum likelihood (ML) tree of *Volvox* sect. *Volvox* based on ITS region of nuclear ribosomal DNA (ITS-1, 5.8S rDNA, and ITS-2) (S1 Table). Bootstrap values from ML (left) and maximum parsimony (right) analyses are shown on the branches.

**Fig 3:**
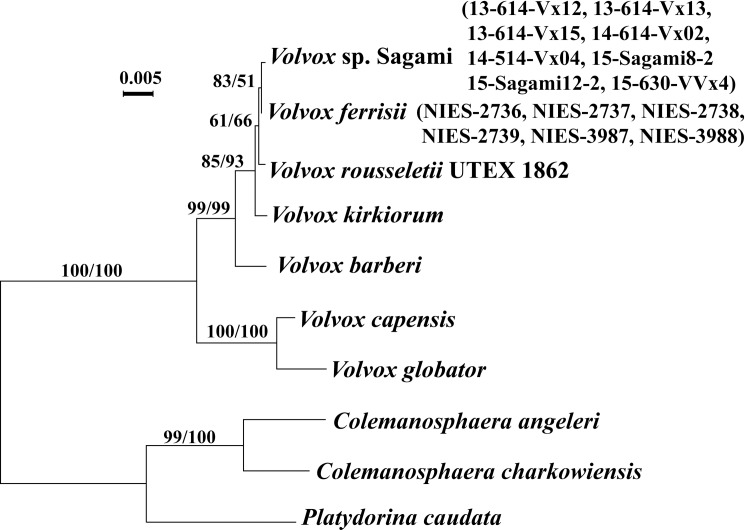
Maximum likelihood (ML) tree of *Volvox* sect. *Volvox* and other colonial Volvocales based on *rbc*L and *psb*C genes (S1 Table). Bootstrap values from ML (left) and maximum parsimony (right) analyses are shown on the branches.

Phylogenetic relationships within *Volvox* sect. *Volvox*, resolved by the ITS rDNA and chloroplast *rbc*L-*psb*C gene trees (Figs 2 and 3) were essentially the same as those of a previous study [9], except for the phylogenetic position of *Volvox* sp. Sagami, which is a sister to *V*. *ferrisii*. These two species exhibited only a single nucleotide difference in ITS rDNA (S2 Fig), and a single difference in 780 base pairs of *psb*C coding regions but no differences in the 1128 base pairs of the *rbc*L genes.

### Group I intron inserted in the *psb*C gene

All *Volvox* sp. Sagami strains examined (S1 Table) contained an insertion of a possible group IA intron in the *psb*C gene after the position corresponding to the 672nd nucleotide of the *Gonium pectorale psb*C gene [19] (accession number AP012494) (S2 Fig). The 2054 base pair long intron had an identical sequence in all *Volvox* sp. Sagami strains except for a single change at position 675 of the insertion (in P6 of the intron secondary structure; S3 Fig) in strains 13-614-Vx13 and 14-614-Vx04. Irrespective of the single nucleotide variance in P6, all *Volvox* sp. Sagami strains exhibited an open reading frame (positions 546–891) that encoded a putative site-specific DNA endonuclease (similar to the putative site-specific DNA endonuclease in the trebouxiophycean green alga *Leptosira terrestris*; YP_001382155) in P6 of the intron.

The nucleotide sequence in P7.1 (positions 1739–1832) of the *Volvox* sp. Sagami group IA intron showed a single homologous nucleotide sequence (a possible group I intron inserted in the *Gonium multicoccum psb*C gene, accession number AB044481) within the Volvocales, based on a discontiguous megablast search (E-value = 3e−12) of the whole intron sequences against the NCBI database.

Introns or insertions are lacking in the 780 base pairs determined in the *psb*C genes of all other *Volvox* sect. *Volvox* species/strains [8, 9] (S1 Table).

### Swimming velocity

**Fig 4:**
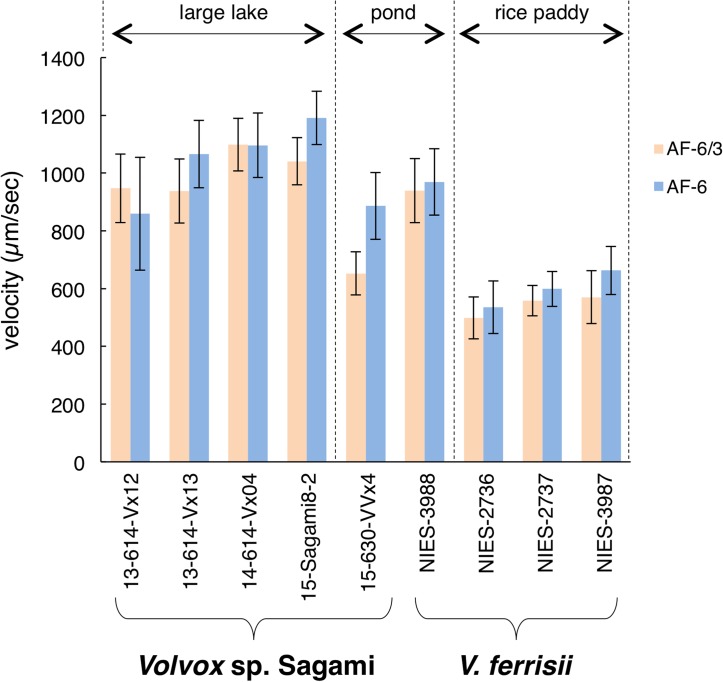
Swimming velocities in various strains of *Volvox* sp. Sagami. and *V*. *ferrisii* Isaka et al. Swimming velocities during phototaxis in each culture were measured by tracing 20 individual *Volvox* spheroids. Two media (AF-6/3 and AF-6) were used for each strain. For statistical tests, see S4 Table.

The swimming velocities during phototaxis of *Volvox* sp. Sagami strains originating from two large lakes (Lake Sagami and Lake Tsukui) were significantly higher than those of the *V*. *ferrisii* strains originating from rice paddies (Fig 4; S4 Table). Two strains of the two species originating from ponds represented an intermediate swimming velocity (Fig 4). There were not large differences in the approximate sizes of spheroids between *Volvox* sp. Sagami from large lakes and *V*. *ferrisii* from rice paddies in cultures examined for swimming velocity (S4 Fig; S5 Table).

## Discussion

*Volvox* sp. Sagami could be clearly identified as the *Volvox* sect. *Volvox*, in having thick cytoplasmic bridges between adult cells and spiny zygote walls [4, 9]. Among the monoecious species of this section, *Volvox* sp. Sagami was similar to *V*. *globator* in having less than 80 eggs or zygotes in a monoecious spheroid (S6 Table). However, *V*. *globator* had flattened anterior somatic cells and blunt zygote wall spines, measuring 3–8 μm long [8], whereas *Volvox* sp. Sagami anterior somatic cells were elongate-ellipsoidal or spindle-shaped, and zygote wall spines were acute and up to 3 μm long (S6 Table).

Although *V*. *kirkiorum* produces monoecious spheroids with a relatively small number (20–80) of eggs or zygotes [8], *Volvox* sp. Sagami differed from *V*. *kirkiorum* according to its short zygote spines and elongated anterior somatic cells (S6 Table). *V*. *kirkiorum* produces spines 5.5–8 μm long and has pear-shaped to ovoid anterior cells [8]. Thus, *Volvox* sp. Sagami may represent an undescribed morphological species. However, *Volvox* sp. Sagami and *V*. *ferrisii* are closely related (Figs 2 and 3), and these two sister lineages showed only a single nucleotide difference in ITS rDNA and no compensatory base changes (CBC) in ITS-1 and ITS-2 rRNA secondary structures (S2 Fig). Thus, these two lineages might be identified as a single species when accepting the species concept based on genetic differences and/or CBC [20]. However, differences in morphological characteristics and habitats were significant. *Volvox* sp. Sagami grows in large lakes and has a small number of eggs (usually 10–20), and short spines on the zygote walls. In contrast, *V*. *ferrisii* is found in rice paddies and produces usually 100–200 zygotes in a monoecious spheroid [8]. In rice paddies, the water is flooded during spring to summer and the water depth is less than 15 cm [21]. The water depth is more than 20 m in Lake Sagami and Lake Tsukui [22]. Therefore, these two Japanese lineages might have diverged rapidly in the two different habitats in Japan. A similar rapid divergence of multiple species was recently reported in dinoflagellates, which are restricted to a few lakes in Russia and Sweden [23].

All of the strains of *Volvox* sp. Sagami showed a group IA intron inserted in the *psb*C genes. However, introns or insertions are completely lacking in the 780 base pairs determined in the *psb*C genes of all other *Volvox* sect. *Volvox* species/lineages [8, 9] (S1 Table). The group IA intron of *Volvox* sp. Sagami *psb*C gene had only a single homologous nucleotide sequence (possible group I intron inserted in the *Gonium multicoccum psb*C gene) within the Volvocales. Thus, the intron should have been transmitted to the common ancestor of all *Volvox* sp. Sagami strains after the divergence of *Volvox* sp. Sagami and *V*. *ferrisii*, suggesting the single origin of the *Volvox* sp. Sagami lineage.

The swimming velocity of *Volvox* sp. Sagami spheroids originating from large lakes was significantly higher than that of *V*. *ferrisii* from paddy fields during phototaxis (Fig 4; S4 Table). Larger spheroids of *Volvox* generally have greater swimming velocities [24]. However, the difference in the swimming velocity in the present study (Fig 4) could not be accounted for by differences in spheroid size between the two habitats, because spheroids in cultures examined for velocity were not significantly different in size among the three habitats (S4 Fig; S5 Table). Thus, we suggest that *Volvox* sp. Sagami has adapted to large water bodies by having high swimming velocities during phototaxis.

Spheroids of *Volvox* sp. Sagami and *V*. *ferrisii* strains originating from ponds exhibited intermediate swimming velocities compared to those from large lakes and rice paddies (Fig 4; S4 Table). Considering that the genetic difference in the nuclear ITS rDNA region and chloroplast *psb*C genes was apparent between *Volvox* sp. Sagami and *V*. *ferrisii* lineages and all *Volvox* sp. Sagami strains have a common *psb*C group IA intron, these two lineages might have at first diverged and adapted to the two markedly different freshwater environments (i.e., large lakes and rice paddies). Then, each lineage might have invaded from a large lake or rice paddy to a pond. Thus, *Volvox* sp. Sagami and *V*. *ferrisii* strains originating from ponds could have secondarily adapted to the new environment. Alternatively, it could be considered that ancestors of *Volvox* sp. Sagami and *V*. *ferrisii* might have grown at first in ponds, and then might have invaded to large lakes and rice paddies. Irrespective of these two alternative hypotheses, swimming velocities of spheroids might have evolved more rapidly than morphological characteristics during the diversification of *Volvox* sp. Sagami and *V*. *ferrisii*.

## Conclusion

Based on the morphological comparison, *Volvox* sp. Sagami apparently represents a new monoecious species of *Volvox* sect. *Volvox* (S6 Table). In addition, *Volvox* sp. Sagami showed genetic differences from its sister (*V*. *ferrisii*) in ITS nuclear rDNA and chloroplast *psb*C, as well as presence of the common insertion of the *psb*C group IA intron in all strains of *Volvox* sp. Sagami. Thus, *Volvox* sp. Sagami represents a lineage that is morphologically and genetically distinct from *V*. *ferrisii*. However, genetic differences between *Volvox* sp. Sagami and *V*. *ferrisii* were small (Figs 2 and 3), falling within a single biological species in other volvocalean algae [20]. Thus, these two lineages may represent a single species that has extensive morphological and ecological diversity, or they may be very closely related different species. Therefore, we did not provide formal description of a new taxon for *Volvox* sp. Sagami. Intercrossing experiments would determine one of these two possibilities. However, such experiments are not practically possible because the two lineages produced monoecious sexual spheroids with sperm packets and eggs. In any case, rapid diversification in morphological and physiological characteristics is apparent in *Volvox* sp. Sagami and *V*. *ferrisii* based on the present study.

## Supporting Information

S1 Fig Two large lakes and a pond from which *Volvox* sp. Sagami was collected in Japan. Original photographs and drawings. A. Lake Sagami (S1 and S3 Tables). B. Lake Tsukui (S1 and S3 Tables). C. Miyaike pond (S1 and S3 Tables).
(DOCX)

S2 Fig The secondary structure of nuclear ribosomal DNA (rDNA) internal transcribed spacer 2 (ITS-2) transcript of *Volvox* sp. Sagami, including the 3’ end of the 5.8S ribosomal RNA (rRNA) and the 5’ end of the LSU rRNA. Secondary structure of nuclear rDNA ITS-2 was drawn using VARNA version 3.9. Note the U-U mismatch in helix II (arrowheads) and the YGGY motif on the 5’ side near the apex of helix III (boldface), common structural hallmarks of eukaryotic nuclear rDNA ITS-2 secondary structures. A single nucleotide difference between *Volvox* sp. Sagami and *V*. *ferrisii* Isaka et al. is shown by a character “G” just outside helix II.
(DOCX)

S3 Fig Secondary structure map of possible group IA intron inserted in *psb*C gene of *Volvox* sp. Sagami. (DOCX)

S4 Fig Comparison of diameters of asexual spheroids between nine strains of *Volvox* sp. Sagami. and *V*. *ferrisii* Isaka et al. in cultures used for measurements of swimming velocity during phototaxis (Fig 4). Two media (AF-6/3 and AF-6) were used for each strain. For statistical tests, see S5 Table.
(DOCX)

S1 Table List of volvocacean species/lineages and strains used in the present phylogenetic analyses (Figs 2 and 3). (DOCX)

S2 Table Primers used for amplification and sequencing of possible group IA intron inserted in *psb*C gene of *Volvox* sp. Sagami. (DOCX)

S3 Table List of strains of *Volvox* sp. Sagami and *V*. *ferrisii* Isaka et al. used in measurements of swimming velocity during phototaxis (Fig 4). (DOCX)

S4 Table Results of analyses of variance (ANOVA) for swimming velocities (x 10 μm/sec) in *Volvox* sp. Sagami and *V*. *ferrisii* Isaka et al. among three habitats and between growth media (AF-6/3 medium and AF-6 medium), using mean value in each of the strains (Fig 4). Based on unweighted-mean ANOVA analyzed by js-STAR version 2.9.9j β < http://www.kisnet.or.jp/nappa/software/star/index.htm >. A = natural habitats. A1 = large lakes. A2 = ponds. A3 = rice paddies. B = growth media.
(DOCX)

S5 Table Results of analyses of variance (ANOVA) for diameters of asexual spheroids (μm) in *Volvox* sp. Sagami and *V*. *ferrisii* Isaka et al. among three habitats and between growth media (AF-6/3 medium and AF-6 medium), using mean value in each of the strains (S4 Fig). Based on unweighted-mean ANOVA analyzed by js-STAR version 2.9.9j β <http://www.kisnet.or.jp/nappa/software/star/index.htm >. A = natural habitats (large lakes, ponds and rice paddies). B = growth media.
(DOCX)

S6 Table Comparison of *Volvox* sp. Sagami and previously described monoecious species of *Volvox* sect. *Volvox*. (DOCX)
