# Supplementary material for: A New Morphological Type of Volvox from Japanese Large Lakes and Recent Divergence of this Type and V. ferrisii in Two Different Freshwater Habitats
Source: PLoS One. 2016 Nov 23;11(11):e0167148. doi: 10.1371/journal.pone.0167148 (PMC5120847; doi:10.1371/journal.pone.0167148)
Supplement: S1 Fig — Original photographs and drawings. A. Lake Sagami (S1 and S3 Tables). B. Lake Tsukui (S1 and S3 Tables). C. Miyaike pond (S1 and S3 Tables). (DOCX) [file pone.0167148.s001.docx]

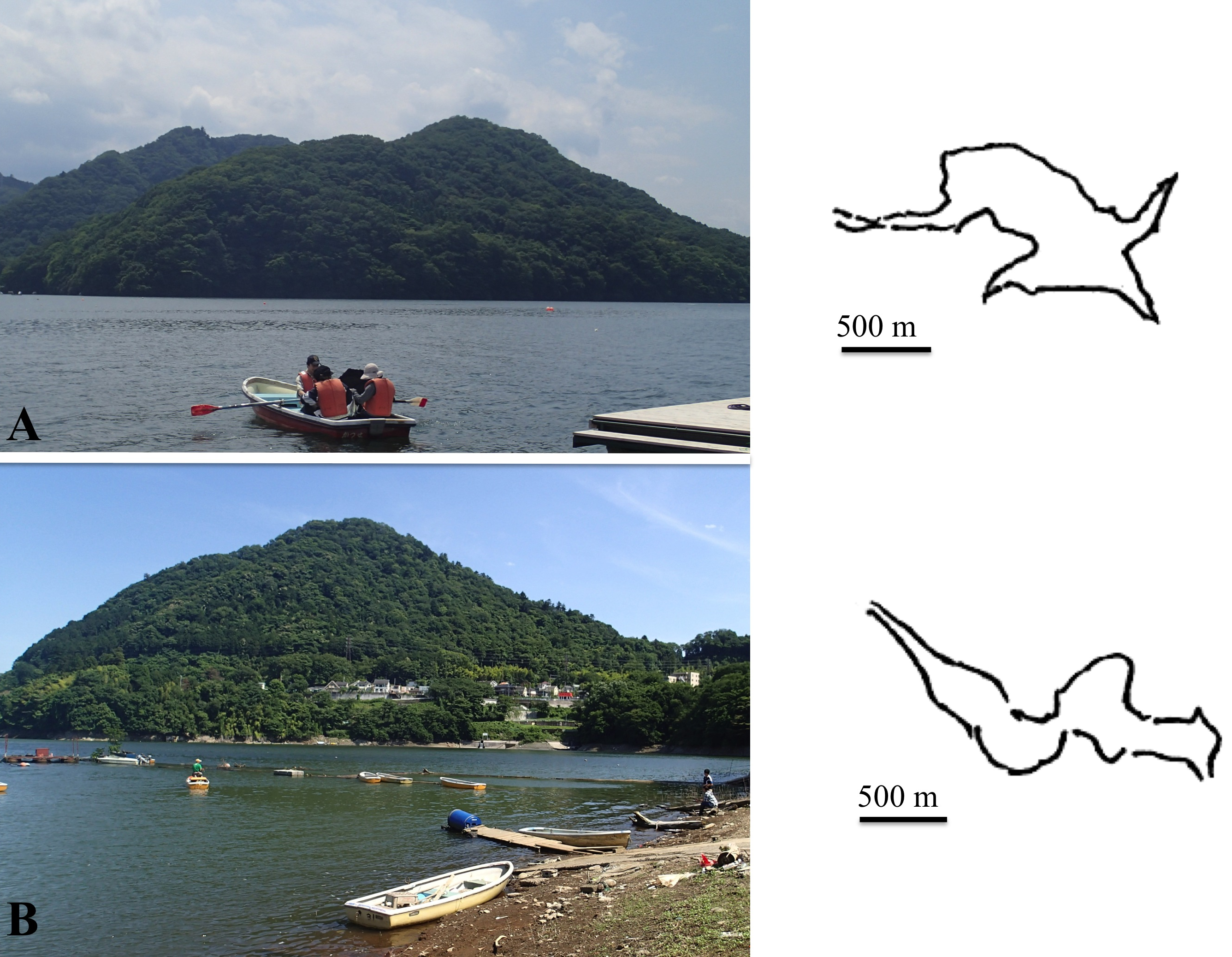


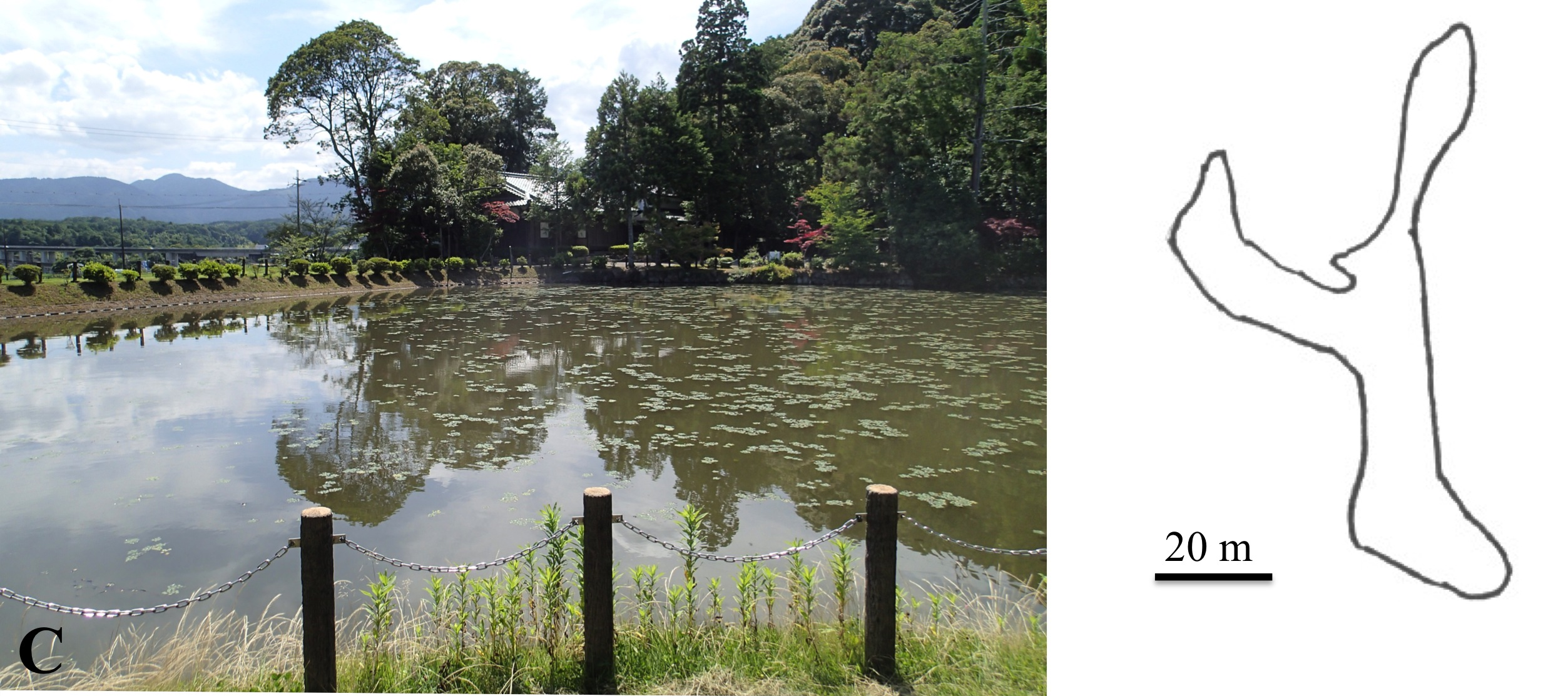


**S1 Fig. Two large lakes and a pond from which *Volvox* sp. Sagami was collected in Japan.** Original photographs and drawings. A. Lake Sagami (S1 and S3 Tables). B. Lake Tsukui (S1 and S3 Tables). C. Miyaike pond (S1 and S3 Tables).
