# Supplementary material for: A New Morphological Type of Volvox from Japanese Large Lakes and Recent Divergence of this Type and V. ferrisii in Two Different Freshwater Habitats
Source: PLoS One. 2016 Nov 23;11(11):e0167148. doi: 10.1371/journal.pone.0167148 (PMC5120847; doi:10.1371/journal.pone.0167148)
Supplement: S2 Fig — Secondary structure of nuclear rDNA ITS-2 was drawn using VARNA version 3.9. Note the U-U mismatch in helix II (arrowheads) and the YGGY motif on the 5’ side near the apex of helix III (boldface), common structural hallmarks of eukaryotic nuclear rDNA ITS-2 secondary structures. A single nucleotide difference between Volvox sp. Sagami and V. ferrisii Isaka et al. is shown by a character “G” just outside helix II. (DOCX) [file pone.0167148.s002.docx]

~~
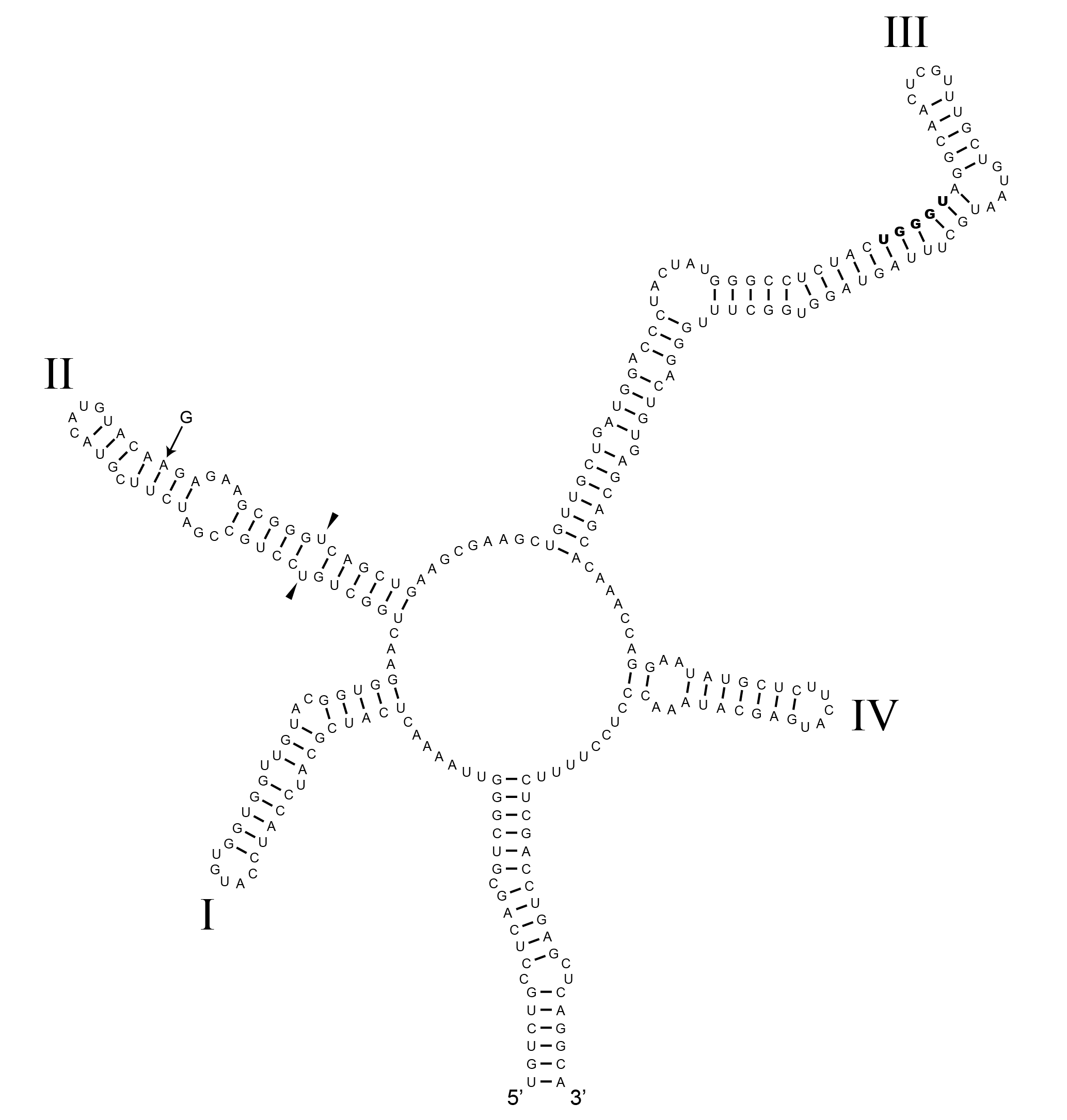
~~

**S2 Fig. The secondary structure of nuclear ribosomal DNA (rDNA) internal transcribed spacer 2 (ITS-2) transcript of *Volvox* sp. Sagami, including the 3’ end of the 5.8S ribosomal RNA (rRNA) and the 5’ end of the LSU rRNA.** Secondary structure of nuclear rDNA ITS-2 was drawn using VARNA version 3.9 [1]. Note the U-U mismatch in helix II (arrowheads) and the YGGY motif on the 5’ side near the apex of helix III (boldface), common structural hallmarks of eukaryotic nuclear rDNA ITS-2 secondary structures. A single nucleotide difference between *Volvox* sp. Sagami and *V. ferrisii* Isaka et al. is shown by a character “G” just outside helix II.

**Reference**

1. Darty K, Denise A, Ponty Y. VARNA: Interactive drawing and editing of the RNA secondary structure. Bioinformatics 2009; 25: 1974–1975. doi: 10.1093/bioinformatics/btp250
