# Supplementary material for: A New Morphological Type of Volvox from Japanese Large Lakes and Recent Divergence of this Type and V. ferrisii in Two Different Freshwater Habitats
Source: PLoS One. 2016 Nov 23;11(11):e0167148. doi: 10.1371/journal.pone.0167148 (PMC5120847; doi:10.1371/journal.pone.0167148)
Supplement: S3 Fig — (DOCX) [file pone.0167148.s003.docx]

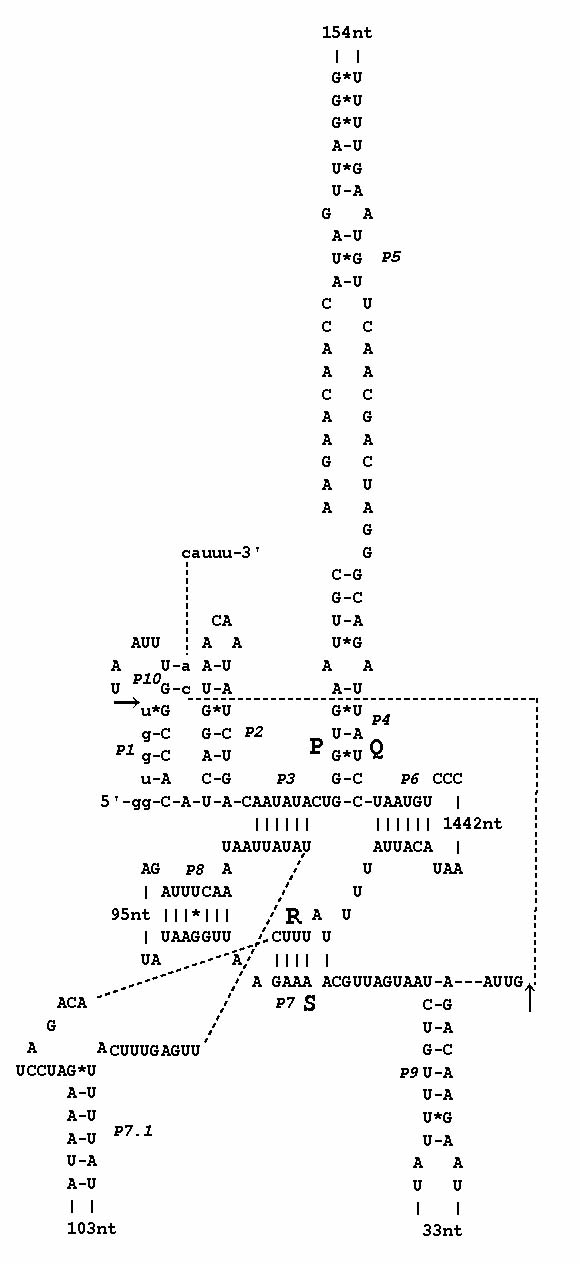


**S3 Fig. Secondary structure map of possible group IA intron inserted in *psb*C gene of *Volvox* sp. Sagami.**
