# Supplementary material for: A New Morphological Type of Volvox from Japanese Large Lakes and Recent Divergence of this Type and V. ferrisii in Two Different Freshwater Habitats
Source: PLoS One. 2016 Nov 23;11(11):e0167148. doi: 10.1371/journal.pone.0167148 (PMC5120847; doi:10.1371/journal.pone.0167148)
Supplement: S4 Fig — Two media (AF-6/3 and AF-6) were used for each strain. For statistical tests, see S5 Table. (DOCX) [file pone.0167148.s004.docx]

**
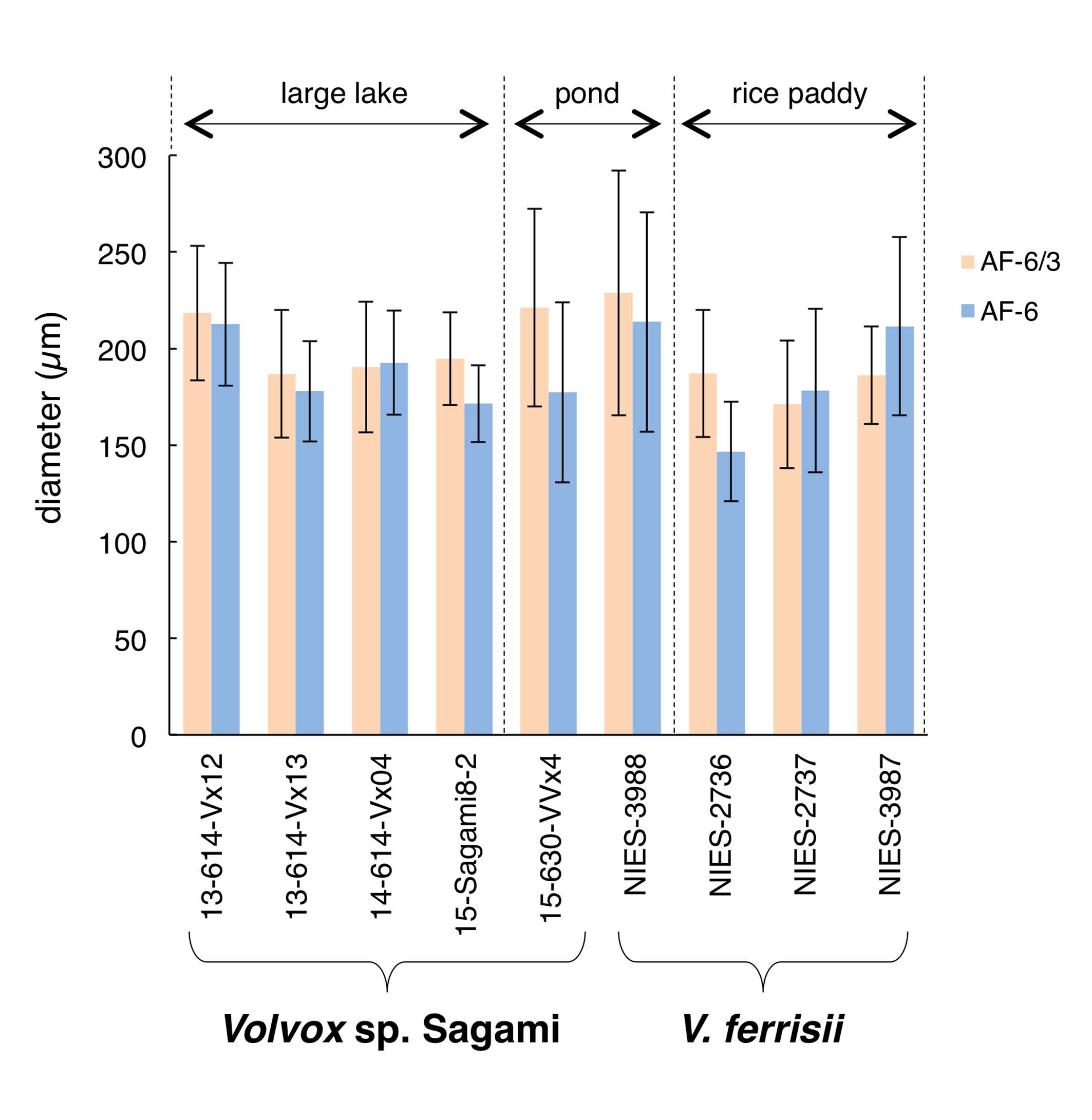
**

**S4 Fig. Comparison of diameters of asexual spheroids between nine strains of *Volvox* sp. Sagami and *V. ferrisii* Isaka et al. in cultures used for measurements of swimming velocity during phototaxis (Fig 4).** Two media (AF-6/3 and AF-6) were used for each strain. For statistical tests, see S5 Table.
