# Supplementary material for: A New Morphological Type of Volvox from Japanese Large Lakes and Recent Divergence of this Type and V. ferrisii in Two Different Freshwater Habitats
Source: PLoS One. 2016 Nov 23;11(11):e0167148. doi: 10.1371/journal.pone.0167148 (PMC5120847; doi:10.1371/journal.pone.0167148)
Supplement: S1 Table — (DOCX) [file pone.0167148.s005.docx]

**S1 Table. List of volvocacean species/lineage and strains used in the present phylogenetic analyses (Figs 2 and 3).**

| Species/lineage | Strain designation | Origin of strain | GenBank/EMBL/DDBJ Accession number | | |
| --- | --- | --- | --- | --- | --- |
|  |  |  | *rbc*L | *psb*C | ITS-1, 5.8S rDNA  and ITS-2 |
| *Volvox* sp. Sagami | 13-614-Vx12 ^a^  (=NIES-4021）  13-614-Vx13 ^a^  (=NIES-4022)  13-614-Vx15 ^a^  (=NIES-4023)  （ | Water sample collected from Lake Sagami, Kanagawa, Japan (water temperature 23°C; pH 8.4; N 35° 36.558’, E 139° 11.280’) in 14 June 2013. | LC191316 ^b^  LC191317 ^b^  LC191318 ^b^ | LC191326 ^b^  LC191327 ^b^  LC191328 ^b^ | LC191308 ^b^  LC191309 ^b^  LC191310 ^b^ |
|  | 14-614-Vx02 ^a^  （=NIES-4024）  14-614-Vx04 ^a^  （=NIES-4025） | Water sample collected from Lake Tsukui, Kanagawa, Japan (water temperature 21°C; pH 8.3; N 35°35.332’, E 139°16.292’) in 14 June 2014. | LC191319 ^b^  LC191320 ^b^ | LC191329 ^b^  LC191330 ^b^ | LC191311 ^b^  LC191312 ^b^ |
|  | 15-Sagami8-2 ^a^  （=NIES-4027）  15-Sagami12-2 ^a^  （=NIES-4028） | Water sample collected from Lake Sagami, Kanagawa, Japan (water temperature 21°C; pH 9.1; N 35° 36.625’, E 139° 11.150’) in 10 June 2015. | LC191321 ^b^  LC191322 ^b^ | LC191331 ^b^  LC191332 ^b^ | LC191313 ^b^  LC191314 ^b^ |
|  | 15-630-VVx4  （=NIES-4026） | Water sample collected from Miyaike Pond, Otsu, Shiga, Japan (water temperature 28°C; pH 8.2; N 35° 08.172’, E 135° 54.440’) in 29 June 2015. | LC191323 ^b^ | LC191333 ^b^ | LC191315 ^b^ |
| *Volvox capensis* | M1-2 | USA | LC033870 | LC033872 | LC034074 |
| *Volvox kirkiorum* | NIES-2740 | Japan | AB663322 | AB663323 | AB663324 |
| *Volvox ferrisii* | NIES-2736  NIES-2737  NIES-2738  NIES-2739  NIES-3987  NIES-3988 | Japan | AB663334  AB663337  AB663331  AB663328  LC191324 ^b^  LC191325 ^b^ | AB663335  AB663338  AB663332  AB663329  LC191334 ^b^  LC191335 ^b^ | AB663336  AB663339  AB663333  AB663330  LC185089  LC185090 |
| *Volvox globator* | SAG 199.80 (=UTEX 955) | USA | D86836 | AB044478 | AB663340 |
| *Volvox barberi* | UTEX 804 | USA | D86835 | AB044477 | AB663341 |
| *Volvox rousseletii* | UTEX 1862 (=NIES-734) | South Africa | D63448 | AB044479 | AB663342 |
| *Colemanosphaera angeleri* | NIES-3382 | Japan | AB905592 | AB905598 |  |
| *Colemanosphaera charkowiensis* | NIES-3383 | Japan | AB905591 | AB905598 |  |
| *Platydorina caudata* | NIES-728 (=UTEX 1658) | USA | D86828 | AB044494 |  |
| ^a^ Established in this study.  ^b^ Sequenced in this study. | |  |  |  |  |
