# Supplementary material for: A New Morphological Type of Volvox from Japanese Large Lakes and Recent Divergence of this Type and V. ferrisii in Two Different Freshwater Habitats
Source: PLoS One. 2016 Nov 23;11(11):e0167148. doi: 10.1371/journal.pone.0167148 (PMC5120847; doi:10.1371/journal.pone.0167148)
Supplement: S2 Table — (DOCX) [file pone.0167148.s006.docx]

**S2 Table. Primers used for amplification and sequencing of possible group IA intron interrupted in *psb*C gene of *Volvox* sp. Sagami.**

| Designation | Position | Sequence (5’–3’) |
| --- | --- | --- |
| VxAr-psbC-In F1 | 497-521 ^a^ | AAGCTATGTACTTCGGTGGTGTTTA |
| VxAr-psbC-In F2 | 200-224 ^b^ | AGCAAAGCAAAATTAGATAAATAAG |
| VxAr-psbC-In R3 ^c^ | 1642-1618 ^b^ | CGACTCGGTTAAATTATGTAATATC |
| VxAr-psbC-In R4 ^c^ | 821-797 ^a^ | GCAATGAAACCCATCATAGATATAG |

^a^ Coordinate numbers from the *Gonium pectorale* *psb*C gene (accession number AP012494).

^b^ Coordinate numbers from the nucleotide sequence of the possible group IA intron (S2 Fig) inserted in the *Volvox* sp. Sagami *psb*C gene (S1 Table).

^c^ Reverse primer.
