# Supplementary material for: A New Morphological Type of Volvox from Japanese Large Lakes and Recent Divergence of this Type and V. ferrisii in Two Different Freshwater Habitats
Source: PLoS One. 2016 Nov 23;11(11):e0167148. doi: 10.1371/journal.pone.0167148 (PMC5120847; doi:10.1371/journal.pone.0167148)
Supplement: S3 Table — (DOCX) [file pone.0167148.s007.docx]

**S3 Table. List of strains of *Volvox* sp. Sagami and *V. ferrisii* Isaka et al. used in measurements of swimming velocity during phototaxis (Fig 4).**

**.**

| Species/lineage | Strain designation | Origin of strain |
| --- | --- | --- |
|  |  |  |
| *Volvox* sp. Sagami | 13-614-Vx12 ^a^（=NIES-4021）  13-614-Vx13 ^a^（=NIES-4022） | Lake Sagami, Kanagawa, Japan (14 June 2013) |
|  | 14-614-Vx04 ^a^（=NIES-4025） | Lake Tsukui, Kanagawa, Japan (14 June 2014) |
|  | 15-Sagami8-2 ^a^（=NIES-4027） | Lake Sagami, Kanagawa, Japan (10 June 2015) |
|  | 15-630-VVx4 ^a^（=NIES-4026） | Miyaike Pond, Shiga, Japan (29 June 2015) |
| *Volvox ferrisii* | NIES-3988 | Pond, Isanuma, Kawagoe-shi.  Saitama, Japan (2 May 2015) ^b^ |
|  | NIES-2736 | Rice paddy, Kawasaki-shi, Kanagawa, Japan (31 January 1984) ^c^ |
|  | NIES-2737 | Rice paddy, Hitachinaka-shi, Ibaraki, Japan (6 July 2004) ^c^ |
|  | NIES-3987 | Rice paddy, Nara, Japan (15 March 2012) ^b^ |
| ^a^ Established in this study (S1 Table). | |  |
| ^b^ From Microbial Culture Collection at the National Institute for Environmental Studies (http://mcc.nies.go.jp/localeAction.do?lang=en). | | |

^c^ From Isaka et al. [1].

**Reference**

1. Isaka N, Kawai-Toyooka H, Matsuzaki R, Nakada T, Nozaki H. Description of two new monoecious species of *Volvox* sect. *Volvox* (Volvocaceae, Chlorophyceae), based on comparative morphology and molecular phylogeny of cultured material. J. Phycol. 2012; 48: 759–767. doi: 10.1111/j.1529-8817.2012.01142.x
