# Supplementary material for: A New Morphological Type of Volvox from Japanese Large Lakes and Recent Divergence of this Type and V. ferrisii in Two Different Freshwater Habitats
Source: PLoS One. 2016 Nov 23;11(11):e0167148. doi: 10.1371/journal.pone.0167148 (PMC5120847; doi:10.1371/journal.pone.0167148)
Supplement: S4 Table — Based on unweighted-mean ANOVA analyzed by js-STAR version 2.9.9j β < http://www.kisnet.or.jp/nappa/software/star/index.htm >. A = natural habitats. A1 = large lakes. A2 = ponds. A3 = rice paddies. B = growth media. (DOCX) [file pone.0167148.s008.docx]

| **S4 Table. Results of analyses of variance (ANOVA) for swimming velocities (x 10 µm/sec) in *Volvox* sp. Sagami and *V. ferrisii* Isaka et al. among three habitats and between growth media (AF-6/3 medium and AF-6 medium), using mean value in each of the strains (Fig 4).** Based on unweighted-mean ANOVA analyzed by js-STAR version 2.9.9j β < http://www.kisnet.or.jp/nappa/software/star/index.htm >. A = natural habitats. A1 = large lakes. A2 = ponds. A3 = rice paddies. B = growth media. | | | | |
| --- | --- | --- | --- | --- |
|  |  |  |  |  |
| S.V | SS | Df | MS | F |
| A | 5986.6603 | 2 | 2993.3301 | 17.92 ** |
| subj | 1001.9583 | 6 | 166.9931 |  |
| B | 264.0032 | 1 | 264.0032 | 5.09 + |
| AxB | 64.0449 | 2 | 32.0224 | 0.62 ns |
| sxB | 310.9583 | 6 | 51.8264 |  |
| Total | 7627.6250 | 17 |  |  |

ns p>0.01; + p<0.10; * p<0.05; ** p<0.01.

| **== Multiple Comparisons by Bonferroni ==** | | |
| --- | --- | --- |
| (MSe= 166.9931, * p<0.05, alpha'= 0.0167) | | |
| [Main Effect of Factor A] | | |
| A | N(Nh) | Mean |
| 1 | 8(5.54) | 103.1250 |
| 2 | 4(5.54) | 86.2500 |
| 3 | 6(5.54) | 57.1667 |
| A1 > A2 * | (BONF= 14.4907) |  |
| A1 > A3 * | (BONF= 12.7796) |  |
| A1 > A2 * | (BONF= 14.4907) |  |

| **== Multiple Comparisons by Holm ==** | | |
| --- | --- | --- |
| (MSe= 166.9931, * p<0.05) | | |
| [Main Effect of Factor A] | | |
| A | N(Nh) | Mean |
| 1 | 8(5.54) | 103.1250 |
| 2 | 4(5.54) | 86.2500 |
| 3 | 6(5.54) | 57.1667 |
| A1 > A2 * | (alpha'= 0.0500) |  |
| A1 > A3 * | (alpha'= 0.0167) |  |
| A2 > A3 * | (alpha'= 0.0250) |  |
