# Supplementary material for: A New Morphological Type of Volvox from Japanese Large Lakes and Recent Divergence of this Type and V. ferrisii in Two Different Freshwater Habitats
Source: PLoS One. 2016 Nov 23;11(11):e0167148. doi: 10.1371/journal.pone.0167148 (PMC5120847; doi:10.1371/journal.pone.0167148)
Supplement: S5 Table — Based on unweighted-mean ANOVA analyzed by js-STAR version 2.9.9j β <http://www.kisnet.or.jp/nappa/software/star/index.htm >. A = natural habitats (large lakes, ponds and rice paddies). B = growth media. (DOCX) [file pone.0167148.s009.docx]

| **S5 Table. Results of analyses of variance (ANOVA) for diameters of asexual spheroids (µm) in *Volvox* sp. Sagami and *V. ferrisii* Isaka et al. among three habitats and between growth media (AF-6/3 medium and AF-6 medium), using mean value in each of the strains (S4 Fig).** Based on unweighted-mean ANOVA analyzed by js-STAR version 2.9.9j β < http://www.kisnet.or.jp/nappa/software/star/index.htm >. A = natural habitats (large lakes, ponds and rice paddies). B = growth media. | | | | |
| --- | --- | --- | --- | --- |
|  |  |  |  |  |
| S.V | SS | df | MS | F |
| A | 2522.1987 | 2 | 1261.0994 | 2.47 ns |
| subj | 3063.9583 | 6 | 510.6597 |  |
| B | 760.1571 | 1 | 760.1571 | 2.93 ns |
| AxB | 558.3526 | 2 | 279.1763 | 1.08 ns |
| sxB | 1556.9583 | 6 | 259.4931 |  |
| Total | 8461.6250 | 17 |  |  |

ns p >0.10.
