# Supplementary material for: A New Morphological Type of Volvox from Japanese Large Lakes and Recent Divergence of this Type and V. ferrisii in Two Different Freshwater Habitats
Source: PLoS One. 2016 Nov 23;11(11):e0167148. doi: 10.1371/journal.pone.0167148 (PMC5120847; doi:10.1371/journal.pone.0167148)
Supplement: S6 Table — (DOCX) [file pone.0167148.s010.docx]

**S6 Table. Comparison of *Volvox* sp. Sagami and previously described monoecious species of *Volvox* sect. *Volvox.***

|  | *Volvox* sp. Sagami | *V. capensis* | *V. globator* | *V. merrillii* | *V. amboensis* | *V. barberi* | *V. kirkiorum* | *V. ferrisii* |
| --- | --- | --- | --- | --- | --- | --- | --- | --- |
| Size of asexual spheroid (µm) | up to 590 | up to 1323 × up to 1357 | 380–500 × 400–575 | 670–890 × 710–925 | 800–1300 | 525–880 × 575–910 | 400–790 × 430–930 ^a^ | 630–1000 × 650–1000 ^a^ |
| Number of cells in asexual spheroid | 2000–10000  (usually 2000–5000) | 2000–23000 | 8000–17000 | 9800–20100 | 30000–50000 | 35000–47800 | 3000–6000 | 5000–8000 |
| Number of gonidia in asexual spheroid | 4–8 | 4–20 (usually 6–10) | 3–17 (usually 4–7) | 3–14 (usually 4–7) | 1–14 (usually 8) | 3–9 | 2–8 (usually 4–6) | 2–8 (usually 3–5) |
| Size of sexual colony (µm) | up to 500 | up to 1292× up to 1335 | 350–480 × 370–510 | 375–650 × 390–690 | up to 2000 | 550–880 × 605–920 | 430–440 × 510–540 ^b^ | 600–800 × 650–900 ^b^ |
| Number of cells in sexual spheroid | 4000–8000 | 3200–6200 or more | 9400–17500 | 12500–23000 |  | 23200–53100 | 1000–6000 | 5000–13000 |
| Number of zygotes (eggs) in sexual spheroid | 5–25 (usually 10–20) | 39–160  [50–220^b^] | 11–70 (usually 20–30) | 45–174 (usually 60–130) | 20–700 (usually more than 200) | 72–251 (usually 100–200) | 20–80 (usually 30–50) | 70–250 (usually 100–150) |
| Number of sperm packets in sexual spheroid | 1–5 | 2–19  [7–35^b^] | 3–7 | 5–8 |  | 4–8 | 4–8 | 3–5 |
| Shape of anterior somatic cells | elongate- ovoid or elongate- ellipsoidal | pear-shaped or ovoid | flattened | flattened (wider than high) | pear-shaped | elongate-ovoid or elongate- ellipsoidal | pear- shaped to ovoid | ellipsoidal to ovoid |
| Diameter of zygotes without spines (µm) | 37–48 | 32–49  [40–53^b^] | 36–55 | 37–40 | 30–37 | 34–40 | 32–38 | 35–40 |
| Shape of spines of zygote | strait with acute apices | straight with rounded or blunt apices | straight with rounded apices | curved with acute apices | curved with acute apices | straight with acute apices | straight with acute apices | straight with acute apices |
| Length of spines  of zygotes (µm) | up to 3 | 4–8  [7–13^b^] | 3–8 | ca. 11 | 5–7 | 3–5.5 | 5.5–8 | 6–8.5 |
| References | the present study | Rich and Pocock [1], Smith [2], Starr et al. [3], Nozaki et al. [4] | Shaw [5], Rich and Pocock [1], Smith [2], Isaka et al. [6] | Shaw [5], Smith [2] | Rich and Pocock [1], Smith [2] | Shaw [5], Smith [2], Isaka et al. [6] | Isaka et al. [6] | Isaka et al*.* [6] |

^a^ Sizes of fully matured spheroids

^b^ *V. capensi*s f. *rhodesiensis*

**References**

1. Rich F, Pocock MA. Observations on the genus *Volvox* in Africa. Ann. S. Afr. Mus. 1933; 16: 427–471, pls. 9–24.

2. Smith GM. A comparative study of the species of *Volvox*. Trans. Am. Microsc. Soc. 1944; 63: 265–310.

3. Starr RC, O'neil RM, Miller CE. L-Glutamic acid as a mediator of sexual morphogenesis in *Volvox capensis*. Proc. Natl. Acad. Sci. USA 1980; 77: 1025–1028.

4. Nozaki H, Ueki N, Misumi O, Yamamoto K, Yamashita S, Herron MD, et al. Morphology and reproduction of *Volvox capensis* (Volvocales, Chlorophyceae) from Montana, USA. Phycologia 2015; 54: 316–320. doi: 10.2216/15-14.1

5. Shaw WR. *Janetosphaera*, a new genus, and two new species of *Volvox*. Philipp. J. Sci. 1922; 20: 477–508, plates 1–5.

6. Isaka N, Kawai-Toyooka H, Matsuzaki R, Nakada T, Nozaki H. Description of two new monoecious species of *Volvox* sect. *Volvox* (Volvocaceae, Chlorophyceae), based on comparative morphology and molecular phylogeny of cultured material. J. Phycol. 2012; 48: 759–767. doi: 10.1111/j.1529-8817.2012.01142.x
